# A Novel Dehydrated Human Umbilical Cord Particulate Medical Device: Matrix Characterization, Performance, and Biocompatibility for the Management of Acute and Chronic Wounds

**DOI:** 10.3390/bioengineering11060588

**Published:** 2024-06-08

**Authors:** Dominique Croteau, Molly Buckley, Morgan Mantay, Courtney Brannan, Annelise Roy, Barbara Barbaro, Sarah Griffiths

**Affiliations:** Research and Development, Stimlabs LLC, 1225 Northmeadow Parkway, Suite 104, Roswell, GA 30076, USA

**Keywords:** umbilical cord, chronic and acute wounds, cellular, acellular, and matrix-like product (CAMP), skin substitute, cellular- and/or tissue-based product (CTP), wound healing, placental tissue, particulate wound device, wound animal model, cell proliferation

## Abstract

Chronic wounds present a significant socioeconomic burden forecasted to increase in prevalence and cost. Minimally manipulated human placental tissues have been increasingly employed and proven to be advantageous in the treatment of chronic wounds, showing improved clinical outcomes and cost-effectiveness. However, technological advances have been constrained by minimal manipulation and homologous use criteria. This study focuses on the characterization of a novel dehydrated human umbilical cord particulate (dHUCP) medical device, which offers a unique allogeneic technological advancement and the first human birth tissue device for wound management. Characterization analyses illustrated a complex extracellular matrix composition conserved in the dHUCP device compared to native umbilical cord, with abundant collagens and glycosaminoglycans imbibing an intricate porous scaffold. Dermal fibroblasts readily attached to the intact scaffold of the dHUCP device. Furthermore, the dHUCP device elicited a significant paracrine proliferative response in dermal fibroblasts, in contrast to fibrillar collagen, a prevalent wound device. Biocompatibility testing in a porcine full-thickness wound model showed resorption of the dHUCP device and normal granulation tissue maturation during healing. The dHUCP device is a promising advancement in wound management biomaterials, offering a unique combination of structural complexity adept for challenging wound topographies and a microenvironment supportive of tissue regeneration.

## 1. Introduction

Chronic wounds broadly describe tissue defects that fail to successfully progress through the normal healing cascade in a timely and organized manner. Approximately 1–2% of the global population will experience a chronic wound in their lifetime, which is shown to directly contribute to a declining quality of life [1,2]. These nonhealing, or hard-to-heal, wounds stem from diverse etiologies and patient comorbidities and create an immense public health burden [1,3]. There exists an unmet medical need for continued wound care innovation to improve outcomes for chronic wounds. Medical device wound dressings have been marked by technological and material advancements in the past century. While initially gauze was once the traditional choice for a wound covering, dressings have evolved over time to include those incorporated with antimicrobials, anti-inflammatory properties, and other elements [4]. More recently, biomaterial-based options, collectively referred to as Cellular, Acellular, and Matrix-like Products (CAMPs; formerly known as skin substitutes and Cellular- and/or Tissue-based Products (CTPs)) have been developed and now exist in an array of offerings [5,6]. Scientific advances in recent decades have enabled the progression from single-component collagen dressings to the emergence of intact extracellular matrix devices with the ultimate goal of closely mimicking complex native human tissue composition and structure [4,5,7,8]. While this evolution of dressings derived from various biomaterial sources brings immense value to the field of wound treatment, immunological barriers to product development often persist, requiring highly involved, and frequently harsh, tissue preparation methods to ensure xenoantigenicity is adequately addressed [9,10].

The regenerative potential of human connective tissue products provides an appealing allogeneic approach to address this challenge, but until now has been restricted due to the existing regulatory framework for human tissue products which limits innovation potential [11]. Human umbilical cord tissue is a distinctive allogeneic biomaterial source that has shown promise for uses in acute and chronic wounds due to the regenerative potential of a complex connective tissue extracellular matrix [12]. The umbilical cord links the placenta and fetus to provide nutrients and oxygen to the growing fetus [13]. A mucoid connective tissue matrix—originally described by Thomas Wharton in 1656—ensures the unrestricted flow of nutrients, imparting distinctive structural properties to resist external forces [14,15]. The intricate structural attributes of this connective tissue matrix that is typically considered biological waste makes for a unique opportunity in regenerative medicine and wound care applications. Herein, we provide a comprehensive matrix composition characterization and in vitro performance evaluation, as well as in vivo biocompatibility and performance, of a newly United States Food and Drug Administration (FDA)-cleared novel dehydrated human umbilical cord particulate (dHUCP) medical device. This study highlights the first human umbilical cord-derived medical device and the first human birth tissue intended for wound management that has received 510(k) clearance from the U.S. FDA [16,17].

## 2. Materials and Methods

### 2.1. Biomaterial Sourcing and Processing

Human birth tissues, consisting of the placenta and umbilical cord, were obtained via donation from healthy, full-term cesarian section births and were screened and tested for risk of communicable diseases in accordance with Food and Drug Administration (FDA) regulations [18] and American Association of Tissue Banks (AATB) standards current at the time of manufacturing. Umbilical cord tissues were separated from the placenta and dissected to remove the umbilical arteries and vein. Tissues were then subjected to proprietary processing, inclusive of a series of rinses designed to remove blood remnants and unwanted constituents while retaining critical desired extracellular matrix components and structural characteristics. The processing steps render a lyophilized, terminally sterilized (SAL 10^−6^ via electron beam irradiation) dehydrated human umbilical cord particulate (dHUCP) medical device (Stimlabs LLC, Roswell, GA, USA). For histological comparisons to raw source material, fresh umbilical cord (f-UC) tissue was aseptically dissected from the maternal disc and briefly rinsed with purified water to remove excess surface blood content. Further, f-UC tissue was cut laterally through the vein wall to open the material for cross-sectional analysis.

### 2.2. Histology and Immunohistochemistry

Histological analysis of native f-UC and the dHUCP device was performed by Premier Laboratory, LLC (Longmont, CO, USA), according to their standard procedures. Samples were fixed in 4% paraformaldehyde and processed for paraffin embedding, then subsequently sectioned and mounted onto charged slides. Glycosaminoglycans (GAGs) and glycoproteins were stained with Alcian Blue (pH 2.5). Slides for Alcian Blue staining were incubated in 3% acetic acid for 2 min prior to being submerged in Alcian Blue 2.5 for 45 min (Anatech LTD, Battle Creek, MI, USA), re-incubated in 3% acetic acid, rinsed in tap water, and placed in Nuclear Fast Red for 10 min. Slides for immunohistochemical staining were incubated in 3.0% hydrogen peroxide followed by epitope retrieval via Proteinase K (Agilent Technologies Inc., Santa Clara, CA, USA), with the exception of slides for Hyaluronic Acid Binding Protein (HABP) staining, which were incubated in Avidin solution (Agilent Technologies Inc., Santa Clara, CA, USA) and Biotin solution (Agilent Technologies Inc., Santa Clara, CA, USA). Slides were then blocked with a serum-free protein solution (Agilent Technologies Inc., Santa Clara, CA, USA) prior to antibody incubation. Primary antibodies against anti-Collagen I (Abcam, Waltham, MA, USA), anti-Collagen III (ThermoFisher Scientific, Waltham, MA, USA), anti-laminin (EnCor Biotechnology, Gainesville, FL, USA), anti-fibronectin (Abcam, Waltham, MA, USA), and biotinylated anti-Hyaluronic Acid Binding Protein (MilliporeSigma, Burlington, MA, USA) were used to stain the slides at room temperature for 30 min (60 min for HABP). Upon conclusion of primary antibody incubation, appropriate HRP-conjugated secondary antibodies (EnVision+; Agilent Technologies Inc., Santa Clara, CA, USA) were applied, followed by application of DAB+ Chromogen solution (Agilent Technologies Inc., Santa Clara, CA, USA). Slides were then rinsed and counterstained in a modified Harris hematoxylin solution (Agilent Technologies Inc., Santa Clara, CA, USA). Stained sections were scanned using the Aperio ScanScope XT imaging system (Leica Biosystems, Deer Park, IL, USA) and ImageScope software version 12.4.6. Scale bars were formatted using ImageJ version 1.54f.

### 2.3. Total Collagen Quantification

Total collagen concentrations were quantified using a Total Collagen Kit (Perchlorate-Free) (Abcam, Waltham, MA, USA) according to manufacturer’s instructions, with some modifications. The dHUCP device material was subjected to alkaline hydrolysis at 120 °C for 1 h without dilution in water or homogenization. Samples were chilled, neutralized with concentrated hydrochloric acid, mixed, and centrifuged. The extracted samples were heated at 65 °C until dried and then reconstituted with the Oxidation Reagent Mix (chloramine T concentrate, oxidation buffer). Samples were incubated for 20 min at room temperature, mixed with Developer solution for 5 min at 37 °C, then further mixed with DMAB Concentrate for 45 min at 65 °C. Sample absorbance was measured at 560 nm. Total collagen concentration was quantified from a standard curve of Collagen I and converted into mg of total collagen per gram of the dHUCP device’s initial dry weight.

### 2.4. Total Sulfated Glycosaminoglycan (sGAG) Quantification

Total sulfated GAG concentrations were quantified using the Blyscan Sulfated Glycosaminoglycan Assay Kit (BioColor Ltd, Carrickfergus, Co Antrim, UK) according to the manufacturer’s instructions, with some modifications. All reagents not provided with the assay kit were sourced from Sigma-Aldrich (St. Lous, MO, USA) unless otherwise stated. The dHUCP device material was digested (5 mg/mL) in papain extraction solution (0.098 M sodium acetate, 0.014 M EDTA, 4.55 mM cysteine HCl (ThermoFisher Scientific, Waltham, MA, USA), 0.44 mM papain, 0.2 M sodium phosphate buffer, pH 6.4) at 65 °C for 3 h, and lysates were diluted with deionized water. Blyscan dye reagent was added to diluted samples that were then gently agitated for 30 min to precipitate out dyed sGAG material. Samples were then centrifuged, dissociation reagent was added to each sample pellet, and samples were transferred to a clear-bottom microtiter plate and read at 656 nm absorbance. The concentration of total sGAG was quantified from a standard curve of chondroitin sulfate and converted into mg of total sGAG per gram of the dHUCP device’s initial dry weight.

### 2.5. Hyaluronan Enzyme-Linked Immunosorbent Assay

The concentration of hyaluronic acid (HA) was measured using an enzyme-linked immunosorbent assay (ELISA) for Hyaluronan (R&D Systems, Minneapolis, MN, USA). The dHUCP device material was digested in papain extraction solution (0.098 M sodium acetate, 0.014 M EDTA, 4.55 mM cysteine HCl, 0.44 mM papain, 0.2 M sodium phosphate buffer, pH 6.4) at 65 °C for 3 h. The homogenates were centrifuged, and the lysates were collected and diluted with assay diluent. Assays were performed according to the manufacturer’s instructions. The dHUCP HA concentrations were normalized to sample dry weights.

### 2.6. dHUCP Device Absorbance Capacity

The contents of a vial of the dHUCP device (1 cc) were weighed pre- and post-hydration with 0.9% normal saline (McKesson, Irving, TX, USA). The absorbance capacity of the dHUCP device was assessed as the change in total mass from its dry state to its hydrated state.

### 2.7. Scanning Electron Microscopy (SEM)

SEM imaging was used to elucidate the ultrastructure of the dHUCP device. Samples were mounted using double-sided carbon tape and imaged with a Hitachi S3700-N scanning electron microscope (Hitachi High-Technologies Corporation, Tokyo, Japan) by Applied Technical Services (ATS; Marietta, GA, USA) according to their standard procedures. Images were taken using the backscatter detector in variable pressure at 60 Pa with an operating voltage of 20 kV. Hitachi S-3700N SEM software (Operation Ver 8.1; Evacuation Ver 2.8) was used for image acquisition. Scale bars were formatted using ImageJ version 1.54f.

### 2.8. In Vitro Cellular Performance Measurements

Normal dermal fibroblasts (WS1 cell line, ATCC, Manassas, VA, USA) were cultured in Eagle’s Minimal Essential Medium (EMEM; ATCC, Manassas, VA, USA) supplemented with 10% fetal bovine serum (FBS; ATCC, Manassas, VA, USA) and 1% penicillin/streptomycin (ATCC, Manassas, VA, USA), according to the manufacturer’s recommendations. For cellular attachment studies, 250,000 cells were seeded onto the dHUCP device. After 6 h, the medium was removed and the seeded device was incubated with the live cell stain Calcein AM (Molecular Probes, Eugene, OR, USA) diluted 1:4 in Dulbecco’s phosphate-buffered saline (DPBS; ATCC, Manassas, VA, USA) at room temperature for 15 min. The stain solution was removed and replaced with fresh DPBS. Pieces of the dHUCP device were placed into new wells after rinsing and were imaged under GFP fluorescence to capture adhered cells while the dHUCP device topography was simultaneously captured via transillumination using an EVOS M5000 imaging system (Invitrogen, Waltham, MA, USA). As a negative control, pieces of the dHUCP device not seeded with cells were subjected to the same incubation and staining procedure. Scale bars were formatted using ImageJ version 1.54f.

For cellular proliferation studies, the dHUCP device and a type I bovine fibrillar collagen powder device were extracted in EMEM supplemented with 2% fetal bovine serum at 37 °C for 6 h with gentle agitation. The soluble fraction was collected and used for proliferation treatment conditions. Cells were seeded at a density of 6250 cells/cm^2^ under standard culture conditions. After 24 h, medium was removed and replaced with a starvation medium (EMEM supplemented with 2% serum) for 6 h—except for the positive control which was maintained in EMEM supplemented with 10% serum for the entire study duration—followed by the application of the prepared dHUCP device and collagen powder extracts prepared as described above, at a final concentration of 2 mg/mL. In tandem, a separate plate subjected to the 6 h starvation was then removed of media and frozen at −80 °C, serving as a ‘Day 0’ reference control. Cells were incubated in the treatment conditions for 72 h, the medium was removed, and the plate was frozen at −80 °C. Upon thawing to room temperature, the proliferation of cells was quantified with CyQUANT Cell Proliferation Assay kit (Molecular Probes, Eugene, OR, USA), according to the manufacturer’s instructions, and read on a plate reader at 480 nm excitation/520 nm emission. Cell-containing wells were background-fluorescence-corrected for media-matched control wells that did not contain cells. The relative proliferation of the 72 h treatment was calculated and reported as the percent change in fluorescence with respect to, or from, the ‘Day 0’ plate fluorescence values for each treatment condition.

### 2.9. Biocompatibility Testing

A porcine full-thickness wound implant study was utilized to assess the biocompatibility of the dHUCP device. The study was conducted by Bridge PTS, Inc. (San Antonio, TX, USA), approved by their Institutional Animal Care and Use Committee, and performed per U.S. Food and Drug Administration Good Laboratory Practice (GLP) regulations set forth in 21 CFR Part 58 and International Organization for Standardization (ISO) 10993-6:2016 tests for local effects after implantation [19]. In preparation for wound creation, three (3) female Yorkshire-cross pigs (35 ± 10 kg) were provided intramuscular injection of Atropine (0.02–0.05 mg/kg), then sedated with Tiletamine–Zolazepam (4.0–6.0 mg/kg) and isoflurane (0.5–5%) mixed with oxygen. The skin was prepared using a chlorhexidine scrub and isopropyl alcohol in alternating fashion three times to mimic the skin preparation in humans.

Full-thickness square (2 cm × 2 cm) wounds were created using a sterile scalpel blade and scissors on the dorsal thorax parallel to each side of the spine, spaced 2–3 cm apart. Hemostasis was confirmed, and wounds were wiped clean to ensure the periwound area was dry prior to treatment. The dHUCP device (2 cc) was applied to wounds on Day 0. Per the device’s instructions for use, the dHUCP device was pre-hydrated with saline, then applied to the base of the wound bed, gently spread evenly across the wound surface up to the wound margins. Each wound was then dressed and monitored regularly. The dHUCP device was not removed nor reapplied for the duration of this study. Pigs were assessed daily for any signs of pain and provided appropriate pain management medication as necessary.

On days 7, 14, and 35, a subset of wounds (10 per timepoint, sampled across all pigs) was sampled for histopathological analyses by excising a strip through the center of the wound to include margins of healthy skin. These timepoints were selected based on guidance in ISO 10993-6:2016 to capture the estimated early and mid-degradation profile timeframe of the product at a clinically relevant implantation site and extend up to or beyond the estimated point of complete degradation. Samples were initially fixed in 10% neutral buffered formalin for at least 24 h, then subsequently fixed in a fresh aliquot of formalin for another 48 h to ensure full formalin penetration. Samples were paraffin-embedded, sectioned, mounted on slides, and stained by StageBio (Frederick, MD, USA) per their standard procedures. Masson’s Trichrome stain was used to evaluate the granulation tissue, namely, to identify regions of residual implant material within the wound sites as well as to characterize the granulation tissue ingrowth, filling, and maturation over time. The prepared stained slides were sent to an independent third party, Inotiv (Fort Collins, CO, USA), for histopathological assessment via light microscopy. Scale bars on representative images were formatted using ImageJ version 1.54f.

Scoring of stained slides was performed according to three parameters: the degree of granulation tissue ingrowth into the dHUCP device material, the degree of granulation tissue that filled the wound bed as the wound healed, and the maturation stage of the granulation tissue in both the superficial and the deep wound bed. Tissue ingrowth into the dHUCP device material was scored as follows: (0) absent; (1) minimal; (2) mild; (3) moderate; (4) marked, as in the degree of change is as complete as possible (i.e., devices not intended to be fully resorbable); or (N/A) material absent/resorbed. Granulation tissue filling of the wound bed was scored as follows: (0) no granulation tissue filling wound; (1) ~1–25% of wound bed filled; (2) ~26–50% of wound bed filled; (3) ~51–75% of wound bed filled; (4) ~76–100% of wound bed filled; or (5) >100% of wound bed filled. Granulation tissue maturation was scored as follows: (0) no collagen deposition; (1) scanty collagen deposition as loose, poorly organized stroma; (2) more notable collagen deposition than Score 1, majority of stroma is loose and poorly organized with collagen fibers predominantly oriented parallel and perpendicular to the skin surface; (3) more notable collagen deposition than Score 2, majority of stroma is dense and organized with collagen fibers oriented parallel to the skin surface; or (4) more notable collagen deposition than Score 3, majority of stroma has the appearance of native dermal collagen.

### 2.10. Statistical Analysis

Statistical analysis was performed using GraphPad Prism 10.1.0 software (San Diego, CA, USA). Specific statistical tests are indicated in the figure legends. A *p* < 0.05 was considered statistically significant.

## 3. Results

### 3.1. Histological Comparison of the dHUCP Device and Its Source Biomaterial

**Figure 1:**
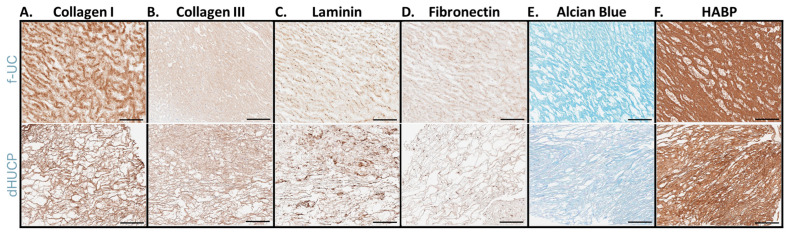
The dHUCP device preserves inherent structural extracellular matrix components and tissue organization mirroring native human umbilical cord. Upper row shows native, fresh umbilical cord (f-UC); lower row shows the dHUCP device for all panels. Sections of each tissue/material were stained for the following connective tissue matrix components: (**A**) anti-Collagen I; (**B**) anti-Collagen III; (**C**) anti-laminin; (**D**) anti-fibronectin; (**E**) proteoglycans and glycosaminoglycans via Alcian Blue; and (**F**) anti-HABP (Hyaluronic Acid Binding Protein). Representative images are shown. Scale bar = 100 microns.

Native umbilical cord connective tissue extracellular matrix (ECM) is known to consist of an array of collagens, glycoproteins, and glycosaminoglycans, including HA [12]. Histological and immunohistochemical staining procedures were performed on native, fresh umbilical cord (f-UC) as well as the umbilical cord-derived device, dHUCP, to compare relative compositional and structural attributes. Both f-UC and the dHUCP device samples contained abundant complex networks of Collagen I (Figure 1A) and Collagen III (Figure 1B). Similarly, fibronectin and laminin immunohistochemical examination of the dHUCP device (Figure 1C,D) showed distributions reflective of the structural organization observed in native f-UC. Alcian Blue stain, performed with pH 2.5 solution, was used to collectively identify the presence of glycosaminoglycans (GAGs) and glycoproteins. Both f-UC and the dHUCP device displayed positive staining via this method (Figure 1E). Further, HA, evaluated via HABP immunohistochemical visualization, was ubiquitous in f-UC and retained in the dHUCP device (Figure 1F).

### 3.2. Extracellular Matrix Profile of the dHUCP Device

**Figure 2:**
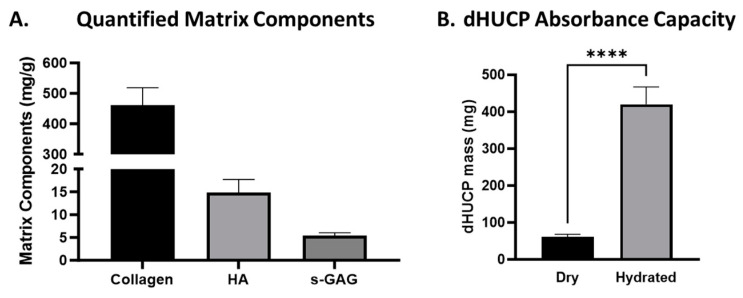
The dHUCP device contains hydrophilic matrix components and readily absorbs moisture. The dHUCP device was assessed for quantified abundance of extracellular matrix proteins previously identified via histology studies. (**A**) Total collagen, total hyaluronic acid (HA), and total sulfated glycosaminoglycan (s-GAG) contents were determined and normalized per mg of dHUCP dry weight. n = 7 per test. (**B**) The ability of the dHUCP device to readily absorb moisture was examined by comparing the device mass from dry to a saline-hydrated state. Paired *t* test; **** *p* < 0.001. Data presented as mean ± SEM, n = 15.

To further understand the composition of the dHUCP device, ECM components were quantified relative to the dry weight of the device (Figure 2A). The total collagen content of the dHUCP device was examined and found to be 460.9 ± 57.5 mg/g, or approximately 46% of the device weight as measured by the Total Collagen kit (Perchlorate-Free) (Abcam). The predominant non-sulfated GAG, hyaluronic acid, accounted for 14.87 ± 2.82 mg/g via ELISA (R&D Systems). Sulfated glycosaminoglycans (GAGs) comprised 5.40 ± 0.66 mg/g of the dHUCP device per the Blyscan Sulfated Glycosaminoglycan Assay kit (BioColor Ltd, Carrickfergus, Co Antrim, UK). Considering the exceptionally hydrophilic nature of these matrix components, the moisture absorbance capacity of the dHUCP device was evaluated. In response to saline hydration, the dHUCP device mass significantly increased more than 6-fold relative to its dry weight (Figure 2B).

### 3.3. Ultrastructure Assessment of the dHUCP Device

**Figure 3:**
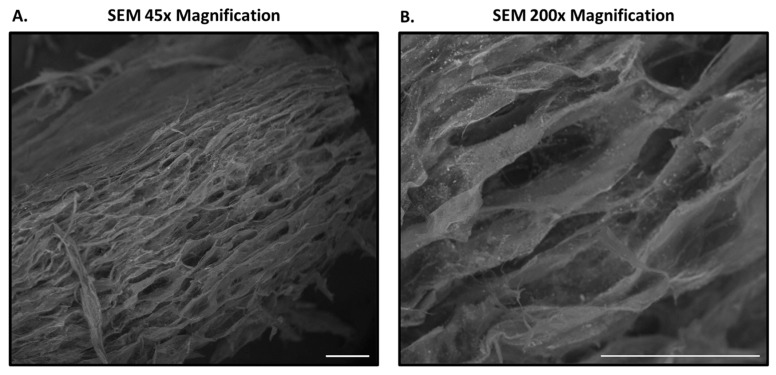
The ultrastructure of the dHUCP device reveals a porous connective tissue matrix. SEM images acquired of a representative dHUCP particle under 45× magnification (**A**) and 200× magnification (**B**) presented a complex, open scaffold structure in the finished, shelf-stable device. Scale bars as indicated in each micrograph. Scale bar = 200 microns for both panels.

In corroborating the histological structural and organizational characterization, Scanning Electron Microscopy (SEM) imaging of the dHUCP device revealed a complex, porous connective tissue matrix (Figure 3). The dHUCP device was observed to consist of a spongy-textured architecture throughout (Figure 3A). Upon closer examination, it is evident that this porous latticework configuration permeates throughout the scaffold, indicative of an intricate, multi-scaled structure (Figure 3B). The overall architecture in these images is consistent with previously published scanning electron micrographs of native human umbilical cord [20].

### 3.4. In Vitro Cellular Performance of the dHUCP Device

**Figure 4:**
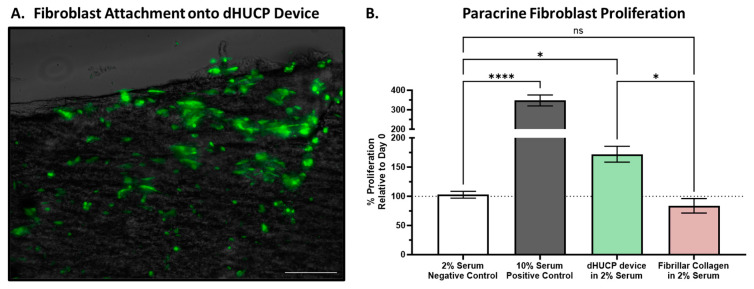
The dHUCP device supports scaffold adherence and induces proliferation of dermal fibroblasts. Dermal fibroblast cells were seeded onto the dHUCP device and allowed to adhere for 6 h. (**A**) Live cells were stained with Calcein AM and visualized under GFP fluorescence with an overlay image of the dHUCP device topography visualized via transillumination. Scale bar = 200 microns. (**B**) Dermal fibroblasts were treated with device extracts (2 mg/mL of 2% serum media) prepared from either the dHUCP device or bovine type I fibrillar collagen powder, and the relative proliferative index was calculated as the growth after 3 days relative to baseline Day 0 values (indicated by the dashed line). The dHUCP device elicited a paracrine proliferative response compared to the 2% serum control, whereas fibrillar collagen was not statistically different from control conditions. Data presented as mean ± SEM, n = 4–6/group, with each n assessed in triplicate. One-way ANOVA with Dunnett’s correction for multiple comparisons; * *p* < 0.05, **** *p* < 0.0001, ns = not statistically significant.

**Figure A1:**
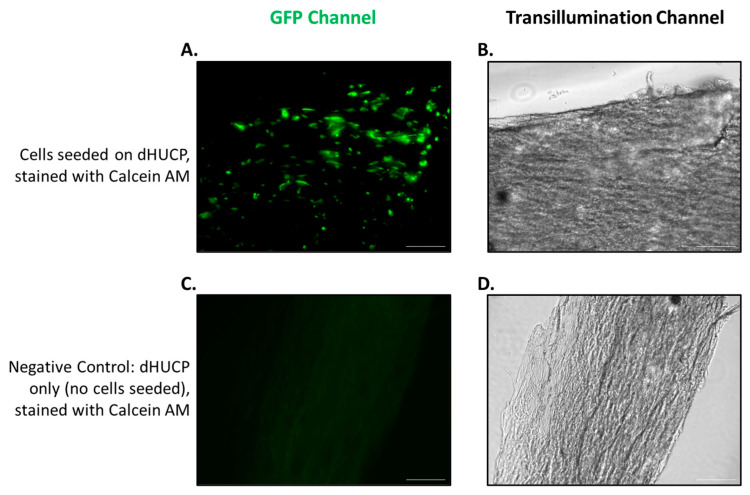
The dHUCP device supports scaffold adherence: supporting images. The dHUCP device with or without dermal fibroblasts seeded, stained with Calcein AM and visualized under GFP fluorescence with an overlay image of the dHUCP device topography visualized via transillumination. Panels (**A**,**B**): the overlay image from Figure 4A has been split, and each channel is shown individually. Panels (**C**,**D**): as a negative comparative control, a sample of the dHUCP device that was not seeded with cells was stained with Calcein AM, with each channel shown individually. Scale bar for all images = 200 microns.

An important attribute of scaffolds in wound management is to support the microenvironment of damaged and deficient tissues by providing a structure conducive for host cellular ingrowth and proliferation. Dermal fibroblast cells were seeded directly onto the dHUCP device in vitro, and after 6 h, the cellular attachment to the device’s scaffold architecture was assessed. As shown in Figure 4A and Figure A1, the seeded cells readily adhered to the dHUCP device, and the fluorescence staining was confirmed to be specific to the live seeded cells. The fibroblast morphology was consistent with adhesion to a scaffold, displaying a flattened morphology with spindle projections elongating out across the dHUCP device. Separately, the ability of the dHUCP device to affect cellular proliferation was assessed by measuring the induction of cell growth in response to extracts of the dHUCP device compared to untreated media-matched controls. In addition, a bovine fibrillar collagen powder was assessed for proliferative effects, as a representative collagen-based CAMP comparator. After 3 days, untreated control fibroblasts displayed minimal to no proliferation (102.6 ± 13.0%) compared to the cell quantity observed at treatment initiation (Day 0) (indicated by the dashed line at the 100% mark on Figure 4B). A significant proliferative effect was observed in the dHUCP device extract-treated fibroblasts of 172.1 ± 33.3% growth compared to the untreated controls (102.6 ± 13.0%; *p* < 0.05). On the other hand, fibrillar collagen powder extracts did not elicit any proliferative phenotype (*p* > 0.05 vs. untreated control) (Figure 4B).

### 3.5. Biocompatibility of the dHUCP Device In Vivo

**Figure 5:**
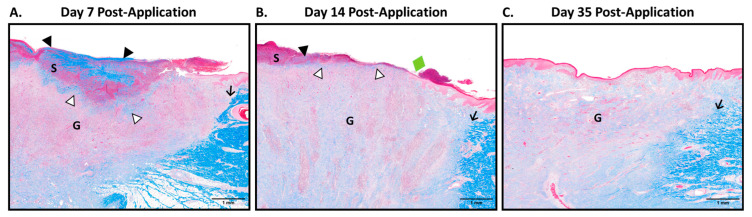
The dHUCP device is biocompatible and completely resorbable in vivo. Full-thickness dermal wounds were created on pigs, and the dHUCP device was applied on Day 0 of wounding. Sample wounds were excised at Days 7, 14, and 35 at study conclusion. Masson’s Trichrome-stained sections were scored for microscopic residual dHUCP device material presence and tissue ingrowth into dHUCP device, as well as granulation tissue filling and maturation. Representative photomicrographs are presented. (**A**) By Day 7, the dHUCP device was noted within the superficial wound bed (open arrowheads), and a thick layer of residual dHUCP device material was also located within the serocellular debris layer (closed arrowheads). (**B**) By Day 14, only half of the excised sample wounds contained detectable residual dHUCP device material, and for the samples that did show residual dHUCP device, it was noted only near the wound surface in the superficial wound bed (open arrowheads) as well as the serocellular debris layer (closed arrowheads). (**C**) By Day 35, no dHUCP device material in any of the sampled wounds was microscopically observed. S = serocellular debris lining the wound surface; G = granulation tissue; arrow = wound margin; green diamond = one edge of re-epithelialization. Scale bars as indicated in each micrograph.

Considering the favorable in vitro findings of cellular attachment and paracrine proliferation induction in dermal fibroblasts, we subsequently investigated the in vivo biocompatibility of the dHUCP device in a porcine full-thickness skin wounding model. Residual dHUCP device material was microscopically detected in all study wound beds sampled at Day 7 and half of wounds sampled at Day 14 and was absent from all wounds sampled on Day 35, indicative of full resorption (Table 1, representative images in Figure 5). The residual material presented as dark blue aggregates on the Masson’s Trichrome stain and was typically located within the superficial wound bed but was also occasionally noted in the mid-wound bed in eight of ten samples at Day 7 and rarely observed in the deep wound bed (one of ten samples at Day 14) (Table 1; Figure 5A,B). By Day 35, no residual dHUCP device was detectable in any of the ten sampled wounds. Tissue ingrowth into the dHUCP device material, as evaluated via histopathologic microscopic analysis, was observed as minimal to mild at Day 7 (average score 1.7 ± 0.5) and moderate at Day 14 (3.0 ± 0.0) in this porcine model. Concordantly, since no residual dHUCP device material was microscopically detected in any of the Day 35 samples, tissue ingrowth was unable to be scored (‘N/A’) (Table 1; Figure 5C).

Granulation tissue filled more than half of each sampled wound bed by Day 7 (with a score of 3.3 ± 0.5) and appeared fully filled by Day 14 (with a score of 4.3 ± 0.5), as evidenced by wounds sampled at that timepoint (Table 2). As the wounds healed, the tissue maturation progression was observed. The deep wound bed exhibited a higher tissue maturation score relative to the superficial wound bed (2.0 ± 0.0 vs. 1.0 ± 0.0 at Day 7; 3.0 ± 0.0 vs. 2.0 ± 0.0 at Day 14), with the superficial wound bed reaching a score of 3.0 ± 0.0 by Day 35 (Table 2).

## 4. Discussion

The goal of this study was to characterize the technical aspects of the first medical device derived from human umbilical cord which has received clearance by the FDA for the management of wounds. The dHUCP device is a CAMP comprising a complex network of connective tissue extracellular matrix proteins, predominantly fibrous collagens interwoven with GAGs including abundant amounts of HA, providing a porous scaffold with a hydrophilic profile. Importantly, the matrix content and organization of the native umbilical cord scaffold are preserved in the dHUCP device, resulting from the intentionally designed, proprietary processing method. In vitro studies further confirmed the importance of the retention of critical components within the dHUCP device, as dermal fibroblasts readily attached to the dHUCP device and proliferated in response to extracts of the dHUCP device. In establishing the biocompatibility profile of the dHUCP device, a porcine full-thickness wounding study was performed, in which the dHUCP device supported tissue ingrowth and granulation tissue maturation during normal wound healing progression. Structural composition, performance, and biocompatibility testing provides a thorough understanding of the first human umbilical cord-derived wound management device.

The porous, open structure of native umbilical cord represented in the dHUCP device is a complex configuration of matrix proteins that not only establish the architecture of the device but also foster a favorable environment for wound healing by virtue of its structural composition and integrity. Wounds require a balance of moisture within the wound bed to ensure a functional wound healing cascade, and loss of this moisture balance can promote a chronic wound environment [21]. The particulate format paired with the absorptive profile of the dHUCP device aids in the balanced moisture management of wounds and is especially applicable for chronic wounds. The particulate format allows for the egress of exudate from the wound bed, and components found in the dHUCP device—such as HA, collagen, and proteoglycans—have been shown to possess hydrophilic properties and are associated with wound healing [22,23,24].

HA possesses a well-established hydrophilic profile shown to have functional benefits in wound moisture management [25,26]. The inclusion of HA in a wound therapy product correlates with better preservation of a balanced, moist environment in a wound bed [26,27,28] as well as increased proliferation of granulation tissue and re-epithelialization [29]. In vivo models have shown that scaffolds loaded with HA promoted moisture retention in wounds and led to accelerated healing compared to scaffolds without HA [30,31]. Congruently, HA contributes to successful wound management through its role in tissue repair and regeneration via various cell signaling pathways [24]. Additionally, the structural contribution of HA to porous scaffold networks has been shown to allow for the diffusion of cells and proteins that facilitate the creation of pathways for cellular migration via chemotaxis [22,32]. More broadly, proteoglycans, which are laden with covalently bonded hydrophilic GAG chains, have been shown to contribute to the wound healing cascade by providing a moist environment conducive to chemotaxis and vascularization [33,34]. In conjunction with the absorbance capacity observed in the dHUCP device, the characteristics of these abundant GAGs, including HA, in the dHUCP device likely play a role in the mechanisms related to wound healing.

Furthermore, the wound exudate in chronic wounds tends to have higher levels of proteases such as matrix metalloproteinases and elastase (which can degrade newly forming tissue) and pro-inflammatory cytokines, causing the wound to remain stagnant in the inflammatory phase of healing [35,36,37]. In fact, some wound dressings have been purposely designed to sequester and/or inactivate proteases as a means to facilitate wound repair [36,38]. The dHUCP device, by virtue of its complex matrix composition, roughly half of which is collagens by weight, may additionally function as a sacrificial substrate source upon which various proteases may act, preserving surrounding healing tissues and simultaneously balancing protease activity. The multifaceted composition of the dHUCP device offers a variety of substrates, including collagens, laminin, and fibronectin, to the various proteases, compared to simple single-component wound dressings.

Scaffolds for wound management may be composed of a single component or may be complex/composite, containing any combination of extracellular matrix components, natural polymers, synthetic or chemically synthesized polymers, and/or bioglass [39,40,41,42]. Regardless of the raw material, there are several key considerations for determining the suitability of a scaffold material beyond its compositional makeup, including biocompatibility; biodegradability; appropriate structural properties for the desired application; a porous scaffold architecture permissive of the movement of cells, nutrients, and waste; and the availability of manufacturing technology for sufficient scale-up [41]. Advances in the field seek to create an ideal wound product that encompasses these qualities [42]. While the use of individual polymers has the advantage of enhanced control over the scaffold constitution, recapitulation of the intricacies of intact human tissue remains challenging.

Along with providing structural support to the wound, complex matrix scaffolds are important vehicles for providing a microenvironment conducive to host cellular migration, proliferation, and differentiation [43,44]. It has been shown that fibroblasts from chronic wounds are more senescent than healthy dermal fibroblasts, making it nearly impossible for them to contribute to healing a chronic wound [45]. The dHUCP device provides a favorable cellular environment, as dermal fibroblasts readily attached to the dHUCP device. While the fibrillar collagen did not allow for comparable cellular attachment studies to be performed in this work due to its fine powder format, by virtue of its simple makeup, it can be postulated that it may be a less ideal substrate for cellular attachment than the dHUCP device, as purified proteins are incapable of adequately mimicking the structural complexity of native connective tissue matrix [7].

Separately, extracts of the dHUCP device elicited a proliferative phenotype in dermal fibroblasts. Notably, the dHUCP device extract was a potent inducer of cellular proliferation, whereas extracts of fibrillar collagen, a commercially ubiquitous matrix-based wound dressing, did not affect the dermal fibroblasts’ growth. Umbilical cord tissue is known to contain an array of matrix- and cell-based components, including both soluble and insoluble factors, which can benefit the progression of tissue repair including collagens, fibronectin, laminins, proteoglycans, hyaluronic acid, and chondroitin sulfates, as well as numerous cytokines and growth factors (reviewed in [12]). Recent studies suggest the mechanism of action for tissue regeneration of wounds can at least in part be strongly mediated by the release or provision of soluble factors [46,47,48,49,50]. Based on the in vitro proliferation results, it can be proposed that native connective tissue matrix-affiliated soluble factors [51,52,53] likely drive the paracrine host cellular response to the dHUCP device and warrant further investigation.

Taken holistically, these structural and performance features of the dHUCP device are complemented by the confirmation of wound resolution and resorption in vivo. Although no pre-clinical model captures all aspects of human wound healing, porcine wound models offer the advantages of anatomical and physiological aspects of dermal wound repair which are representative of human skin and healing mechanisms [54,55]. Additionally, the porcine model allowed for the reduction to the lowest possible number of animals due to the large skin surface area characteristic of adult pigs. Maximizing the quantity of wound sites per animal allowed for a study capable of reasonably providing meaningful data interpretation necessary for GLP studies and aligned with the Replace, Reduce, and Refine guidelines supported by the US FDA [56]. As expected, due to the resorbable particulate nature of the dHUCP device, residual device material was observed to be incorporated into all layers of the wound bed. Wounds treated with the dHUCP device achieved appropriate granulation tissue filling, tissue ingrowth, and expected maturation of granulation tissue, which are all metrics of normal healing progression. These findings provide in vivo insight on the dHUCP device’s mechanisms that can support wound management, including the combination of matrix composition and device configuration. Future studies to assess the relative contributions of these various attributes of the dHUCP device would benefit the development of next-generation wound therapy products.

Due to its inherent structural, performance, and biocompatibility characteristics, the dHUCP device is aptly designed for hard-to-heal wounds. In a non-sponsored third-party case, an umbilical cord particulate product was used to treat a chronic, crusted ulceration on the parietal aspect of the scalp that had persisted for one year [57]. The patient had failed to respond to corticosteroid treatment for 8 weeks prior to treatment with the umbilical cord particulate product. After 4 weekly applications of the cord particulate product, the wound was 45% smaller. After 8 weekly treatments, near-complete resolution was achieved, and no wound recurrence was observed during the 9-month follow-up period. This peer-reviewed case report supports that a device composed of umbilical cord particulate not only functions to support acute wound healing in an animal model but can also initiate stalled phases of the wound healing cascade, thus allowing for the proper healing process to proceed in chronic wounds, especially those presenting with challenging wound topographies.

A unique feature of the dHUCP device is that it is derived from human birth tissue, materials immunologically tolerated in clinical use without requiring immunosuppressive treatment, thus generally considered immune-privileged [58,59,60]. While xenogeneic CAMPs are commercially plentiful, the often harsh solutions and/or physical processes required to combat antigenicity can result in stripping desirable native components and reducing extracellular matrix structural integrity [61,62]. In contrast, the dHUCP device retains its compositional complexity, as evidenced in this work, and successfully passed the full panel of biocompatibility requirements in accordance with ISO 10993: ‘Biological Evaluation of Medical Devices Part 1: Evaluation and Testing’ for US FDA 510(k) medical device clearance (listed in [17]). Furthermore, clinical testing with the dHUCP device further supported the safety evaluation of the device in human subjects (Human Repeat Insult Patch Test, Skin Prick Test) as part of the 510(k) clearance of the dHUCP device [17]. As a whole, the dHUCP device avoids the typical pitfalls of biomaterial-based wound dressings, offering the first allogeneic wound device and pioneering a path towards a more optimal wound management paradigm.

With innovative technological advancements, such as the dHUCP device, further studies can demonstrate how this connective tissue matrix can benefit a wider range of indications, such as third-degree burns, as well as inform the utility of additional device formats. Notably, comprehensive characterization of the moisture management capabilities in tissue-based CAMPs are lacking, despite the broadly understood benefits of maintaining a proper moisture balance to facilitate wound healing [21,63]. Furthermore, while hydrophilic matrix components, such as those found in the dHUCP device, are generally considered conducive to moisture balance, tissue granulation, and other processes intrinsic to proper wound healing [12,24], future studies elucidating specific roles of these components in the context of wound healing will be informative. As the first human birth tissue-derived medical device cleared for wound management as well as the first human umbilical cord medical device to achieve 510(k) clearance, the dHUCP device is a novel advancement poised to make a promising impact on a diverse etiology of wounds.

## 5. Patents

Translucent, dehydrated placental tissue and methods of producing and using the same [64]. 

## Figures and Tables

**Table 1:** dHUCP device material tissue ingrowth scores, device presence, and device distribution within wound beds over time in a porcine full-thickness wound model.

Parameter	Day 7								Day 14								Day 35							
	n	Score						Mean ± SD	n	Score						Mean ± SD	n	Score						Mean ± SD
		0	1	2	3	4	N/A			0	1	2	3	4	N/A			0	1	2	3	4	N/A	
Tissue ingrowth into dHUCPdevice (score)	10	0	3	7	0	0	0	1.7 ± 0.5	10	0	0	0	5	0	5	3.0 ± 0.0	10	0	0	0	0	0	10	N/A
Residual dHUCP device material presence		P (10 of 10 wounds)								P (5 of 10 wounds)								A						
Residual dHUCP device material location		S (10 of 10 wounds) M (8 of 10 wounds) D (0 of 10 wounds)								S (5 of 10 wounds) M (0 of 10 wounds) D (1 of 10 wounds)								N/A						

P = present; A = absent; S = superficial wound bed; M = mid-wound bed; D = deep wound bed; SD = standard deviation; N/A = not applicable; n = 10 wounds per time point distributed across 3 pigs.

**Table 2:** Granulation tissue filling and maturation within wound beds over time in a porcine full-thickness wound model.

Parameter	Day 7		Day 14		Day 35	
	n	Mean ± SD	n	Mean ± SD	n	Mean ± SD
Granulation tissue filling of the wound bed (score 0–5)	10	3.3 ± 0.5	10	4.3 ± 0.5	10	4.0 ± 0.0
Granulation tissue maturation in superficial wound bed (score 0–4)		1.0 ± 0.0		2.0 ± 0.0		3.0 ± 0.0
Granulation tissue maturation in deep wound bed (score 0–4)		2.0 ± 0.0		3.0 ± 0.0		3.0 ± 0.0

SD = standard deviation; n = 10 wounds per time point distributed across 3 pigs.

## Data Availability

The data presented in this study are available upon reasonable request from the corresponding author.
